# Asymmetry in sleep spindles and motor outcome in infants with unilateral brain injury

**DOI:** 10.1111/dmcn.15244

**Published:** 2022-04-20

**Authors:** Viviana Marchi, Riccardo Rizzi, Päivi Nevalainen, Federico Melani, Silvia Lori, Camilla Antonelli, Sampsa Vanhatalo, Andrea Guzzetta

**Affiliations:** ^1^ Department of Developmental Neuroscience IRCCS Stella Maris Foundation Pisa Italy; ^2^ Department of Neuroscience, Psychology Drug Research and Child Health NEUROFARBA, University of Florence Florence Italy; ^3^ Department of Clinical Neurophysiology Children's Hospital, HUS Diagnostic Center, Clinical Neurosciences, Helsinki University Hospital and University of Helsinki Helsinki Finland; ^4^ Neuroscience Department, Children's Hospital Meyer University of Florence Florence; ^5^ Neurophysiology Unit, Neuro‐Musculo‐Skeletal Department University Hospital Careggi Florence Italy; ^6^ Department of Clinical Neurophysiology, BABA Center Children's Hospital, Neuroscience Center, HiLIFE, Helsinki University Hospital and University of Helsinki Helsinki Finland; ^7^ Department of Clinical and Experimental Medicine University of Pisa Pisa Italy

## Abstract

**Aim:**

To determine whether interhemispheric difference in sleep spindles in infants with perinatal unilateral brain injury could link to a pathological network reorganization that underpins the development of unilateral cerebral palsy (CP).

**Method:**

This was a multicentre retrospective study of 40 infants (19 females, 21 males) with unilateral brain injury. Sleep spindles were detected and quantified with an automated algorithm from electroencephalograph records performed at 2 months to 5 months of age. The clinical outcomes after 18 months were compared to spindle power asymmetry (SPA) between hemispheres in different brain regions.

**Results:**

We found a significantly increased SPA in infants who later developed unilateral CP (*n*=13, with the most robust interhemispheric difference seen in the central spindles. The best individual‐level prediction of unilateral CP was seen in the centro‐occipital spindles with an overall accuracy of 93%. An empiric cut‐off level for SPA at 0.65 gave a positive predictive value of 100% and a negative predictive value of 93% for later development of unilateral CP.

**Interpretation:**

Our data suggest that automated analysis of interhemispheric SPA provides a potential biomarker of unilateral CP at a very early age. This holds promise for guiding the early diagnostic process in infants with a perinatally identified brain injury.

**What this paper adds:**

- Unilateral perinatal brain injury may affect the development of electroencephalogram (EEG) sleep spindles.
- Interhemispheric asymmetry in sleep spindles can be quantified with automated EEG analysis.
- Spindle power asymmetry can be a potential biomarker of unilateral cerebral palsy.

## What this paper adds

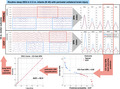

**Abbreviations**
MCA: Middle cerebral artery
SPA: Spindle power asymmetry

A common neuroanatomical substrate for the development of cerebral palsy (CP) is early structural brain damage, which compromises the growth and organization of neuronal networks during early infancy, leading to the wide range of clinical CP phenotypes.^1^ Neonatal stroke is a well‐known cause of adverse neurodevelopmental outcomes, including unilateral CP. The global prevalence of neonatal stroke is around 1 in 3000 live births, and over half of the affected children develop long‐term disabilities, including unilateral CP.^2^, ^3^ Growing evidence supports the importance of early identification of infants at high risk for CP, as early detection might allow for timely therapeutic interventions aiming to exploit the neuroplastic potential of the developing brain.^4^, ^5^

Sleep spindles are hallmarks of non‐rapid eye movement sleep, representing a stereotypical, widely coordinated neuronal activity in the thalamocortical and cortico‐cortical networks.^6^, ^7^, ^8^ The relative ease of sleep spindle detection with electroencephalography (EEG) makes it an attractive candidate for an early functional biomarker of neurodevelopmental network disorders such as CP. Indeed, studies on adults after hemispheric stroke suggest that functional asymmetry between hemispheres, and in particular a reduction in sleep spindles over the lesioned hemisphere, is correlated with stroke severity and outcome.^9^, ^10^, ^11^, ^12^, ^13^, ^14^ These effects, however, have not been studied in infants where rapid functional and structural development of brain networks adds another dimension to be inspected: the sleep spindles themselves emerge around the second month of life, which is long after the occurrence of the structural brain lesion.^7^, ^8^

In the present work, we set out to study whether spatial reorganization of sleep spindle activity is present after unilateral neonatal brain injury. We hypothesize that a marked interhemispheric asymmetry of sleep spindles' amplitude would be specific for a pathological network reorganization after unilateral brain injury that leads to unilateral CP.

## METHOD

### Study design and participants

A multicentre cohort (*n*=40; 19 females, 21 males) was retrospectively collected from three hospitals in Italy (Azienda Ospedaliera‐Universitaria di Pisa [*n*=8), Azienda Ospedaliera‐Universitaria Meyer [*n*=7], and Azienda Ospedaliera‐Universitaria Careggi [*n*=4]) and the Children's Hospital of the Helsinki University Hospital in Finland (*n*=21). Patients were identified from a 9‐year period (1st January 2011 to 31st December 2019) from each local EEG archive. Details of the study population are reported in Table 1. The median gestational age at birth was 40.1 weeks (interquartile range [IQR] 1.18; range 36.6–42.3). The median age at recording was 3.0 months postterm (IQR 1.5; range 1.5–5.0). The median age at the last follow‐up was 24 months postterm (IQR 18; range 18–117).

**Table 1:** Demographics

	All groups, *n*=40	Unilateral CP, *n*=13	Typical outcome, *n*=27
**Females (%)**	19 (48)	6 (46)	13 (48)
**Lesion side, left (%)**	22 (55)	8 (61)	14 (52)
**Median (IQR)** **age at EEG (mo)**	3.0 (1.5–5.0)	3.0 (1.5–5.0)	3.0 (2.0–5.0)
**Median (IQR)** **age at follow‐up (mo)**	24.0 (18.0–117.0)	35.9 (19.0–117.0)	22.5 (18.0–84.0)
**Median (IQR)** **gestational age (wks)**	40.1 (36.6–42.3)	40.4 (37.6–41.9)	40.0 (36.6–423)

Abbreviations: CP, cerebral palsy; IQR, interquartile range; EEG, electroencephalogram.

Inclusion criteria were: (1) a diagnosis of unilateral brain injury such as focal ischemic or haemorrhagic injury confirmed on neonatal magnetic resonance imaging (MRI); (2) at least one sleep‐EEG recording between weeks 8 and 20 after term age, with at least 5 minutes of clear N2‐non‐rapid eye movement sleep; and (3) clinical follow‐up of at least 18 months.

Exclusion criteria were: (1) preterm birth (<36wks gestational age); (2) bilateral brain injury; (3) a known other brain malformation; (4) isolated hypoxic–ischemic brain injury; (5) epidural or subdural hematoma; (6) intraventricular haemorrhage without signs of parenchymal venous infarction.

### Standard protocol approvals, registrations, and patient consents

For the Italian cohort, the relevant local institutional research review board approved the study protocol and ethics approval was obtained by the Tuscany Paediatrics Ethics Committee (SPACE2020; nr.187/2020) and for the Finnish cohort, the study protocol was approved by the Institutional Research Review Board at Helsinki University Hospital Medical Imaging Centre, Helsinki University Hospital. In both countries, a waiver of consent was granted because of the retrospective and observational nature of the study.

### MRI acquisition and classification

All infants underwent standard diagnostic brain MRI during the first 2 weeks of life using a 1.5T scanner (Helsinki: Philips Intera Achieva, Philips Medical Systems, Best, the Netherlands; Santa Chiara Hospital and Meyer Children Hospital: GE, Signa Horizon, Milwaukee, WI, USA) or a 3T scanner (Helsinki: Siemens Magnetom Skyra, Siemens Healthcare GmbH, Erlangen, Germany). Acquisition protocols slightly differed among the clinical centres, but all included at least T1‐, T2‐, and diffusion‐weighted images. For the Italian cohort, retrospective classification of brain lesions was performed by authors (VM, RR) based on the available MRI. For the Finnish cohort, as 11 infants were the same as in Nevalainen et al.,^15^ we used the scores assigned in that study; for the rest of the infants, MRI data were classified from the radiology reports based on the identification of the different vascular territories as previously reported by Kirton et al. and Goveart et al. and recently adapted by Nevalainen et al.^15^, ^16^, ^17^ Classification of stroke subtypes included the following: (1) proximal middle cerebral artery (MCA) territory including lateral lenticulostriate arteries, leading to infarction of basal ganglia and distal MCA territory; (2) distal MCA territory, involving the M1 segment, sparing basal ganglia, and lateral lenticulostriate arteries; (3) posterior trunk territory including parietal and posterior temporal lobes; (4) anterior trunk territory including frontal lobes and anterior temporal lobes; (5) other territory of the distal segments of the MCA not classifiable as posterior trunk or anterior trunk; (6) other arterial territories not classifiable as MCA territory; (7) territory of the lateral lenticulostriate arteries (mainly putamen, caudate body, and posterior limb of internal capsule); (8) medullary venous territory infarction extending into the periventricular white matter with a relative spare of basal ganglia and cortex; (9) periventricular venous infarction secondary to intraventricular haemorrhage; and (10) intracerebral lobar haemorrhage.

### EEG acquisition

EEG recordings of the identified patients were retrospectively collected from the hospital archives. All the patients had undergone a full video‐EEG recording of at least 40 minutes, including sleep and wake phases, with at least 5 minutes of clear N2‐non‐rapid eye movement sleep. Three different EEG systems were used in these hospitals (Meyer Children Hospital and Santa Chiara Hospital: Brain Quick, Micromed, Italy; Careggi General Hospital: Galileo, EBNeuro, Italy; Helsinki University Hospital: NicoletOne, Natus, Middleton, WI, USA). Recordings were sampled at 256Hz, and the EEG signals were acquired using paediatric EEG caps with at least 10 Ag/AgCl electrodes located at Fp1, Fp2, F3, F4, C3, C4, T3, T4, O1, O2 according to the international 10–20 system. All visual and computational analyses were performed on a standard bipolar montage: Fp1‐C3, C3‐O1, Fp1‐T3, T3‐O1, Fp2‐C4, C4‐O2, Fp2‐T4, T4‐O2. A low‐pass filter with a cut‐off of 70Hz and a high‐pass filter with a cut‐off of 0.3Hz was applied.

Sleep epochs were visually identified (by VM, PN, RR, FM), and the N2‐non‐rapid eye movement sleep epochs extracted from every EEG recording. The periods with obvious artefacts were removed by visual inspection and the channels with poor signal were excluded from the analysis. EEG data epochs were then exported into .edf format and imported into MATLAB (2018B, MathWorks, Natick, MA, USA) using customized routines.

### Automated spindle detection and spindle power analysis

Spindle events were selected using an automatic spindle detector employing a customized algorithm in MATLAB (written by author VM) based on previously published procedures.^6^, ^10^, ^18^, ^19^ For each event on every channel, we then calculated the spindle oscillation frequency and the mean spindle power. The algorithms can be found in an open GitHub repository (https://github.com/vivi‐mar/EEGspindles_SPA). According to the maturational and spatial patterns,^18^ we then categorized the spindles depending on their respective frequency ranges as slow (11–13Hz), fast (13–15Hz), or full‐band (11–15Hz). The mean spindle power was then computed for each of these frequencies and for every bipolar channel: fronto‐central, fronto‐temporal, centro‐temporal, centro‐occipital, and temporo‐occipital.

Asymmetry in spindle power was regionally calculated as the ratio between the spindle spectral power of the lesioned and non‐lesioned hemispheres. As a result, 15 separate spindle power asymmetry (SPA) indexes were obtained depending on the region and the frequency assessed: fronto‐central‐full‐SPA, fronto‐temporal‐full‐SPA, centro‐temporal‐full‐SPA, centro‐occipital‐full‐SPA, temporo‐occipital‐full‐SPA, fronto‐central‐slow‐SPA, fronto‐temporal‐slow‐SPA, centro‐temporal‐slow‐SPA, centro‐occipital‐slow‐SPA, temporo‐occipital‐slow‐SPA, fronto‐central‐fast‐SPA, fronto‐temporal‐fast‐SPA, centro‐temporal‐fast‐SPA, centro‐occipital‐fast‐SPA, temporo‐occipital‐fast‐SPA.

Details of the spindle selection procedure and steps of power analysis and interhemispheric asymmetry are reported in Figure S1.

### Motor outcome

As part of the standard care for infants with brain injury, all patients were included in the neurodevelopmental follow‐up programme performed by the paediatric neurologists of the four tertiary centres. Motor outcome (classified as typical motor outcome and unilateral CP) was collected after retrospective review of the medical record (authors VM, PN, CA, RR) of the last follow‐up visit (range 1y 6mo–9y 10mo; minimum 18mo).

### Statistical analysis

Statistical analysis was performed with IBM SPSS statistics software v.23.0 (IBM SPSS Statistics, Version 23.0. Armonk, NY, RRID: SCR_002865). Demographic characteristics were compared for infants with and without unilateral CP using the χ^2^ test for binary variables. For comparisons of continuous variables between the outcome groups, we used the Mann–Whitney *U* test given that the data did not present a normal distribution (Shapiro–Wilk test, as the sample was below the 50 participants). We chose a *p*‐value lower than 0.05 as the level of statistical significance in a two‐tailed test.

SPA indexes which differed significantly between the two groups (*p*<0.05) were then entered in a binary logistic regression model to predict the outcome. The forward conditional method was run by setting the criterion value to enter the model in the forward selection as 0.05, and the criterion value to leave the model in the backward elimination as 0.1. By default, the starting model was the constant model.

Finally, we calculated the sensitivity, specificity, positive and negative predictive values, and area under receiver operating characteristic curve for the SPA index that was most accurate for predicting CP based on the logistic regression model. We applied the Grubb's test to check for the presence of any significant outliers and rebooted the analysis after their removal.

### Data availability

All relevant data are within the paper and Appendix S1. Raw EEG data supporting the findings of this study are available from the authors in an anonymized format, upon reasonable request from a qualified investigator and approval by the ethics boards of the corresponding institutions for purposes of replicating procedures and results. The algorithms used for the spindle analysis are available from an open GitHub repository (https://github.com/vivi‐mar/EEGspindles_SPA).

## RESULTS

In total, 13 out of 40 neonates developed unilateral CP. There were no significant differences in the demographic variables between infants with versus without unilateral CP (Table 1).

Neonatal MRIs were classified according to the stroke patterns. Most of the lesions (*n*=27) occurred in the territories of the MCA: 10 infants had lesions involving the proximal tract of the MCA, five were in the distal branches, three in the anterior trunk, seven in the posterior trunk, two in the lateral lenticulostriate arteries territory. In two infants, lesions occurred in other arterial territories than MCA. Of the remaining infants, six presented periventricular venous infarction, three intracerebral haemorrhage, and two periventricular haemorrhagic infarction. In our sample, all infants who developed unilateral CP had unilateral brain injury involving the MCA territory, while those having a positive motor outcome were distributed across all MRI classification groups.

### SPA and outcome

**Figure 1:**
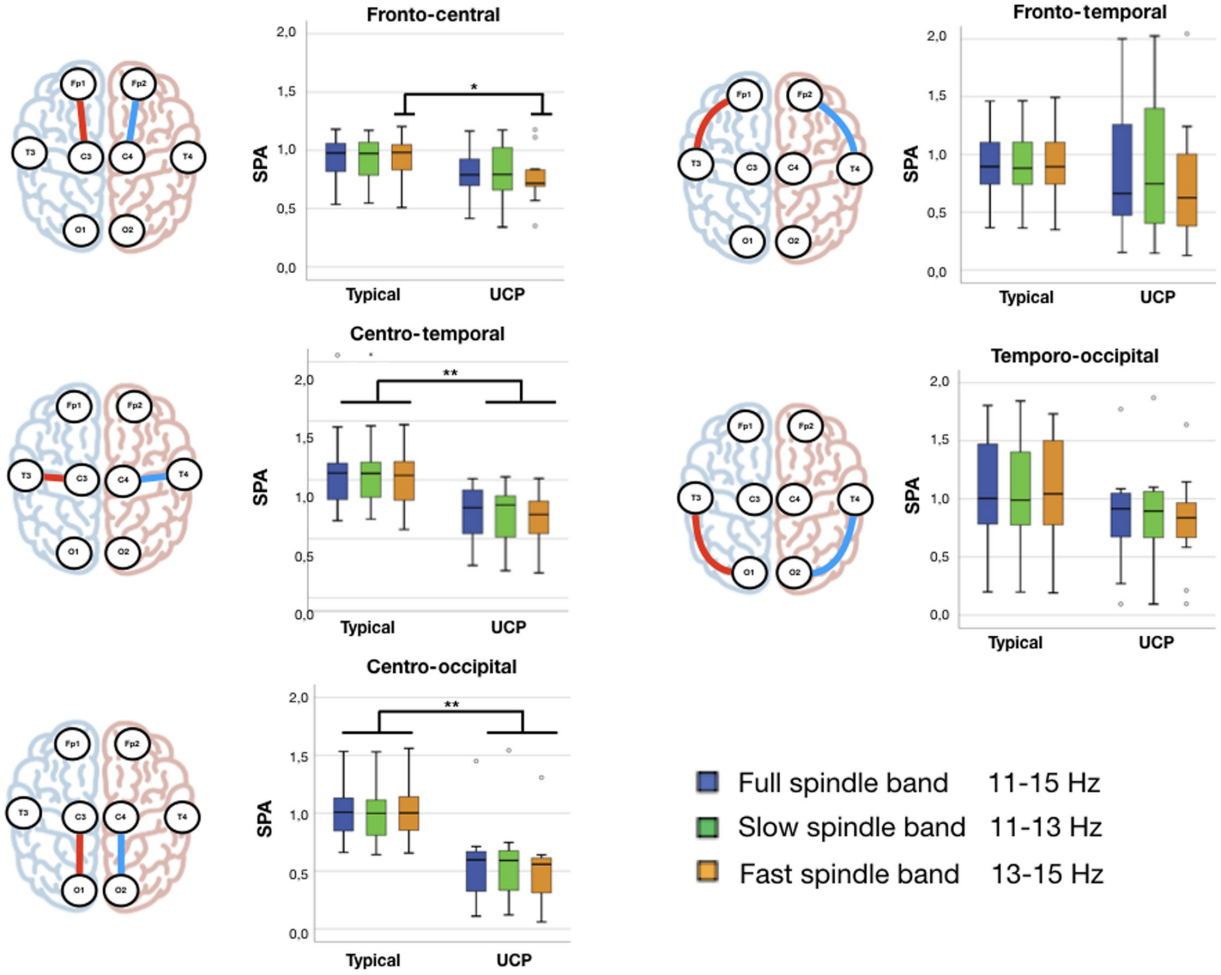
Graphical representation of regional spindle power asymmetry (SPA) indexes. Colour boxplots are grouped based on different outcomes (UCP: unilateral cerebral palsy; typical: typical outcome). The boxplots represent median and interquartile range; whiskers represent 95% confidence intervals; outliers are depicted as circles. For each region a schematic drawing of the bipolar electrode placement is reported.

We first examined whether early SPA significantly differed between the clinical outcome groups. The infants with eventual unilateral CP had a greater SPA in their wideband spindles (11–15Hz) in the centro‐temporal and centro‐occipital regions (centro‐temporal‐full‐SPA *p*<0.001; centro‐occipital‐full‐SPA *p*<0.001). Frequency‐wise analysis showed the group difference to be frequency specific: the slow spindles (11–13Hz) differed between clinical outcome groups in the same centro‐temporal and centro‐occipital derivations (centro‐temporal‐slow‐SPA *p*<0.001; centro‐occipital‐slow‐SPA *p*<0.001), while the fast spindles (13–15Hz) showed a group difference also in the fronto‐central derivations (fronto‐central‐fast‐SPA *p*=0.014; centro‐temporal‐fast‐SPA *p*<0.001; centro‐occipital‐fast‐SPA p<0.001). Table 2 and Figure 1 summarize results from all comparisons. There was, however, no significant association between SPA and the patterns of MRI‐detected structural brain injury.

**Table 2:** Regional spindles power asymmetry indices

Asymmetry		Median	IQR	Mann–Whitney *U*test	*p*
FC‐full‐SPA	Typical	0.98	0.28	111.00	0.064
	UCP	0.79	0.26		
FT‐full‐SPA	Typical	0.89	0.38	151.00	0.493
	UCP	0.66	0.90		
**CT‐full‐SPA**	**Typical**	**1.06**	**0.31**	**61.00**	**<0.001**
	**UCP**	**0.76**	**0.45**		
**CO‐full‐SPA**	**Typical**	**1.01**	**0.31**	**33.00**	**<0.001**
	**UCP**	**0.60**	**0.39**		
TO‐full‐SPA	Typical	1.00	0.73	135.00	0.252
	UCP	0.92	0.43		
FC‐slow‐SPA	Typical	1.97	0.32	125.00	0.151
	UCP	0.79	0.40		
FT‐slow‐SPA	Typical	0.88	0.37	157.00	0.608
	UCP	0.75	1.06		
**CT‐slow‐SPA**	**Typical**	**1.05**	**0.32**	**62.00**	**<0.001**
	**UCP**	**0.79**	**0.44**		
**CO‐slow‐SPA**	**Typical**	**1.00**	**0.33**	**39.00**	**<0.001**
	**UCP**	**0.59**	**0.42**		
TO‐slow‐SPA	Typical	1.00	0.66	137.00	0.276
	UCP	0.90	0.43		
**FC‐fast‐SPA**	**Typical**	**0.98**	**0.26**	**91.00**	**0.014**
	**UCP**	**0.72**	**0.18**		
FT‐fast‐SPA	Typical	0.89	0.39	123.00	0.135
	UCP	0.62	0.73		
**CT‐fast‐SPA**	**Typical**	**1.04**	**0.35**	**46.00**	**<0.001**
	**UCP**	**0.71**	**0.36**		
**CO‐fast‐SPA**	**Typical**	**1.00**	**0.32**	**25.00**	**<0.001**
	**UCP**	**0.56**	**0.35**		
TO‐fast‐SPA	Typical	1.04	0.73	132.00	0.217
	UCP	0,84	0.43		

Note: *p*‐values less than 0.05 are statistically significant and marked in bold.

Abbreviations: IQR, interquartile range; FC, fronto‐central; SPA, spindle power asymmetry; UCP, unilateral cerebral palsy; FT, fronto‐temporal; CT, centro‐temporal; CO, centro‐occipital; TO, temporo‐occipital.

**Figure 2:**
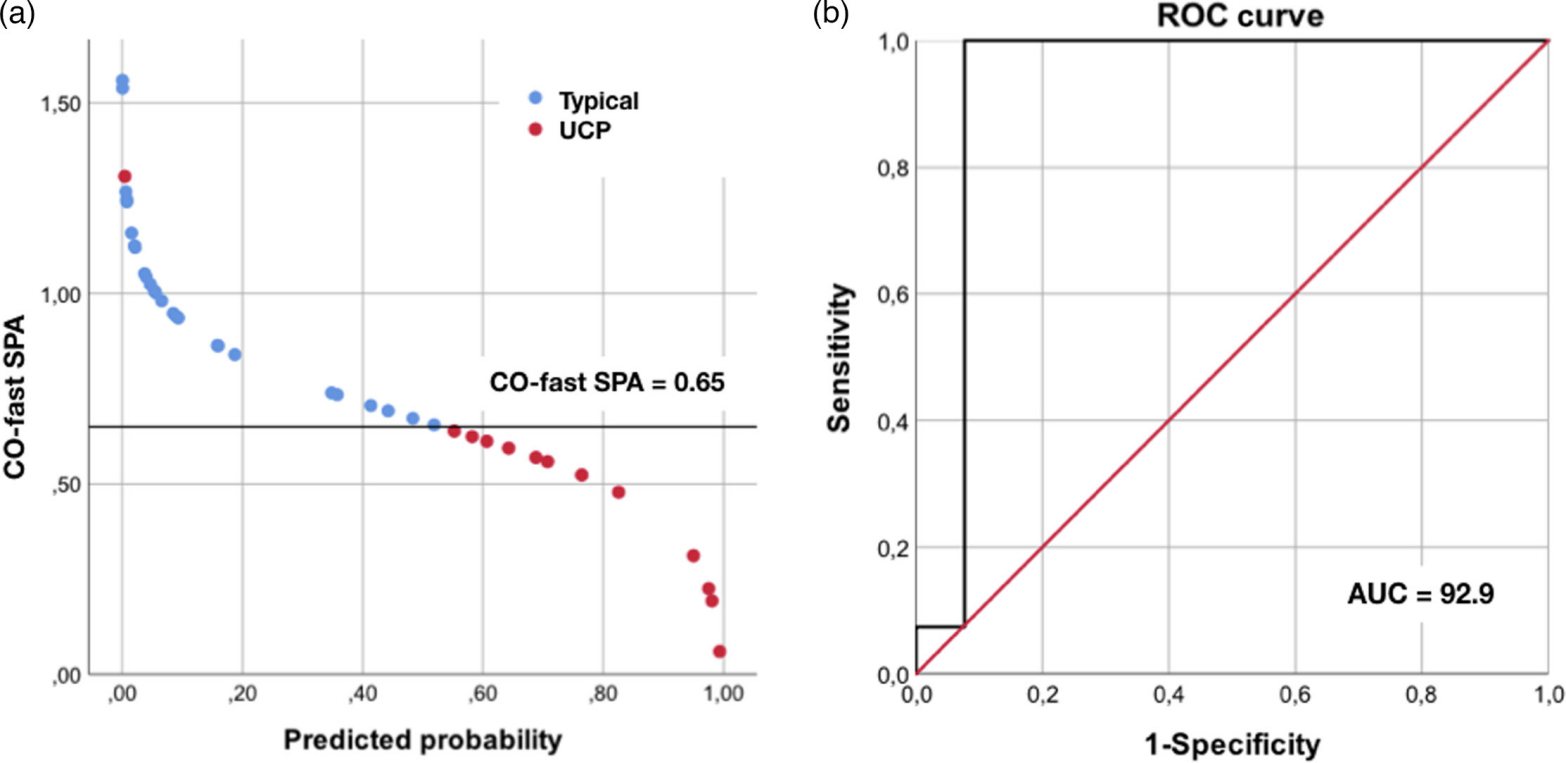
Unilateral cerebral palsy (UCP) prediction by centro‐occipital fast sleep spindle power asymmetry (CO‐fast‐SPA). (a) Distribution of CO‐fast‐SPA values according to the corresponding predicted probability of developing UCP. Each circle represents one participant; colours represent different outcomes: red for UCP and blue for typical motor outcome. The selected cut‐off for CO‐fast‐SPA is represented by the horizontal black line. (b) Receiver operating characteristic (ROC) curve and relative area under receiver operating characteristic (AUC) curve of the CO‐fast SPA for UCP prediction

We then tested the ability of SPA to predict unilateral CP at the individual level by entering all the seven SPA indexes that significantly differed between the centro‐occipital outcome groups in a binary logistic regression model. An asymmetry between fast spindles over centro‐occipital regions (centro‐occipital‐fast SPA) was found to provide the best prediction (centro‐occipital‐fast SPA Wald=08.61, *p*=0.003), explaining 44.6% (Nagelkerke *R* ^*2*^) of the variance in outcome and correctly classifying 95.0% of cases, if considered alone in the regression model. The area under the receiver operating characteristic curve for centro‐occipital‐fast SPA was 0.93 (standard error: 0.07, asymptotic significance: 0.00, asymptotic 95% confidence interval: 0.79–1.00; see Figure 2). An empiric cut‐off level for SPA at 0.65 gave a positive predictive value of 100% (sensitivity 100.0%) and a negative predictive value of 93.3% (specificity 92.3%) for later development of unilateral CP.

We finally checked the presence of outliers by applying the Grubb's test (two‐sided *p*=0.05) to the whole population and no outlier was selected. When we applied the Grubb's test by splitting the population in the two different outcome groups, it showed the presence of significant outliers in the group with unilateral CP: one outlier was identified when tested for temporo‐occipital and for centro‐occipital SPA indexes. No outlier was detected in the group with typical motor outcome. After removing the outliers, we confirmed our results. For centro‐occipital‐SPA, the statistical difference between the outcome groups was even stronger (centro‐occipital‐full‐SPA, *p*<0.000; centro‐occipital‐slow‐SPA, *p*<0.000; centro‐occipital‐fast‐SPA, *p*<0.000); for temporo‐occipital‐SPA, the removal of outliers confirmed the non‐different distribution of SPA indexes between the two outcome groups. The subsequent steps of the analytical process confirmed the selection of centro‐occipital‐fast SPA as the best predictor.

## DISCUSSION

Our results show that unilateral brain injury in newborn infants links to interhemispheric asymmetry of sleep spindles in those infants that later will develop unilateral CP. Our work extends prior research by showing that sleep spindles, a functional bedside measure of thalamocortical networks, may be a clinically useful biomarker in neurophysiological assessments at an early age. These findings corroborate recent studies on adults suggesting that sleep spindles may provide an endogenous functional biomarker of neuromotor outcomes after vascular brain injury.^10^, ^13^

Sleep spindles emerge during the second month after term birth and are clearly seen over the central areas which involve the crucial networks implicated in the neuromotor functions affected in CP.^7^, ^18^ The brain networks sustaining sleep spindles show rapid activity‐dependent growth and organization during the early development of brain circuitry.^20^, ^21^ In our study, asymmetry of the fast spindles over the centro‐occipital regions proved to be the best predictor of later motor impairment. This is not surprising as during the first year of life the fast spindles (13–15Hz) peak over the central regions, while the slow spindles (11–13Hz) become more dominant over the frontal regions after the second year of life.^18^ Overall these findings confirm that the integrity of motor‐sensory feedback over the somatosensory cortex could play a critical role in later motor development.

Sleep spindles are sustained by and are hence considered to reflect the integrity of thalamocortical circuitry.^7^, ^21^ Sleep spindle activity is thought to be involved in sleep‐dependent brain plasticity, memory consolidation, and learning,^7^, ^20^, ^21^, ^22^ but also to be particularly sensitive after pathological events, such as focal brain injuries. Studies in adult patients showed that the occurrence and synchrony of spindles are greatly reduced after an injury affecting the sensorimotor cortex,^9^, ^11^, ^14^ the thalamic nuclei, or the thalamocortical projections.^6^, ^13^, ^23^ Moreover, after hemispheric stroke, amplitude asymmetry of spindles significantly correlates with long‐term motor outcome of adult patients,^9^, ^14^ indicating that poststroke sleep changes could be used as early biomarkers of atypical motor development.^10^, ^13^ Our study extends these findings to neonatal brain injuries, a time window when spindles are yet to emerge, suggesting that the spindle asymmetry reflects an underlying disruption in thalamocortical networks, leading to the long‐term neurodevelopmental consequence of unilateral CP. Recent studies on structural imaging are in line with the key role of thalamocortical pathways in the emergence of both unilateral and bilateral types of CP after perinatal stroke.^24^, ^25^, ^26^ Moreover, abnormalities in the thalamocortical projections are even a stronger predictor of motor outcome compared to findings of the corticospinal tract.^24^

Early prediction of unilateral CP is crucial for identifying infants to early intervention programmes, as well as to adequately support and counsel caregivers. Current clinical recommendations support the combination of structural brain MRI with the General Movements Assessment to estimate the risk of unilateral CP before the 5th month of age.^4^ The sleep spindles assessment could provide an independent predictive measure to this age range. Previously, clinical qualitative interpretations of neurophysiological recordings were used to predict outcome after perinatal brain injury.^15^, ^27^, ^28^, ^29^ In contrast, the hereby introduced SPA indexes offer an objective and fully automated measure to be extracted from the clinical EEG recordings, which are often routinely performed during epileptological follow‐up of perinatal stroke. As such, SPA indexes may support clinicians in the selection of high‐risk infants after unilateral brain injury, providing a quantitative measure of risk of unilateral CP. Further studies are needed to determine whether SPA indexes provide information over and above the current diagnostic standards (neonatal brain MRI and neurological assessment). Nevertheless, SPA indexes might still offer an opportunity for early assessment of unilateral CP risk in areas where MRI is not easily available, like low‐ and middle‐income countries.

Our study has some limitations that may affect the practical conclusions. First, this work is based on retrospective data collection from specialized medical centres, which might lead to selection bias towards higher‐risk populations; however, the incidence of unilateral CP in our cohort was comparable to the existing literature.^30^ Second, the retrospective study design does not allow a formal assessment of predictive performance or a standardized clinical protocol in the diagnostic thresholds. For instance, early motor assessments (i.e. General Movements Assessment)^4^ or identical neuroradiological protocols were not available for all individuals which limits a thorough comparison to alternative risk stratifications that are currently used in the clinical workups. Third, the size of the patient cohort is limited, and much larger cohorts are needed to establish definitive diagnostic thresholds, prediction performance, and generalization across data sets. Finally, the spindle detector algorithm was customized for this study as a standard part of the methodological development. While it cannot explain the key findings (the SPA group differences), it should be acknowledged that more detailed neurophysiological interpretations of the present results would benefit from a detailed technical validation of the spindle detector in larger cohorts. While any of these issues are unlikely to challenge the overall conclusions in our work, future prospective studies with multicentre settings are needed to accurately define the diagnostic added value of SPA in the clinical context.

In conclusion, the automatically computed measure of interhemispheric sleep SPA holds promise as an early, easy to obtain, and totally automated biomarker for predicting unilateral CP. Should these results be confirmed, SPA could be a valuable tool for the follow‐up of infants at neurodevelopmental risk.

## Supporting information


**Appendix S1:** Spindle extraction pipeline.

**Figure S1:** Sleep spindle detection and spindle power asymmetry procedure.

## Data Availability

The data that support the findings of this study are available from the corresponding author upon reasonable request.
