# Supplementary material for: Asymmetry in sleep spindles and motor outcome in infants with unilateral brain injury
Source: Dev Med Child Neurol. 2022 Apr 20;64(11):1375–82. doi: 10.1111/dmcn.15244 (PMC9790667; doi:10.1111/dmcn.15244)
Supplement: Supplementary file 1 — Appendix S1: Spindle extraction pipeline. [file DMCN-64-1375-s002.docx]

**SUPPLEMENTARY MATERIAL**

**Spindle extraction pipeline.** Spindles were detected for each bipolar channel by power and duration criteria applied on a band-pass filtered signal (11-15 Hz; roll-off 3 dB at 10 and 16 Hz; Chebyshev Type II filter; for details see D’Atri et al 2018)^18^. We applied Hilbert transform to each channel to compute the instantaneous amplitude of the filtered signal (see Sarasso et al. 2014)^19^; spindle events were detected by setting two amplitude thresholds defined on the mean amplitude of every channel: 1. Spindles peak threshold: set at mean + 3 standard deviations (SD); 2. spindles start/end threshold: set at the mean amplitude of each channel. Adjacent events occurring within the 0.5 s were merged; only events with a duration of between 0.5 s and 10 s were further considered spindles.

For every detected event we obtained the spindle oscillation frequency, calculated as the maximal power within the spindle frequency range of the spectrogram using short-time Fourier transform (±2 s around a spindle detection, 1-second windows, 99% overlap and a resolution of 0.2 Hz); according to Andrillon et al., 2011.^6^ Based on the spindle oscillation frequency, we then divided the selected spindles between “fast”, if the selected frequency was between 13 and 15 Hz and “slow”, if the selected frequency was between 11 and 13 Hz, according to D’Atri et al, 2018.^18^

The extended algorithms can be found in the open GitHub repository (<https://github.com/vivi-mar/EEGspindles_SPA> ). See Figure S1 for details.
