# Supplementary material for: Asymmetry in sleep spindles and motor outcome in infants with unilateral brain injury
Source: Dev Med Child Neurol. 2022 Apr 20;64(11):1375–82. doi: 10.1111/dmcn.15244 (PMC9790667; doi:10.1111/dmcn.15244)

Figure S1. Sleep Spindle detection and Spindle Power Asymmetry (SPA) procedure. A) I. representative raw sleep EEG trace (filters 0.3– 70 Hz) showing NREM 2 phase activity detected from the bipolar montage. II. and III. Steps of the spindle detection procedure (amplitude and duration criteria): the sleep-EEG trace in I. was band-pass filtered between 11 and 15 Hz (−3 dB at 10 and 16 Hz) using a 2nd order Chebyshev filter. The instantaneous amplitude of this signal was computed via the Hilbert transform (red trace). A detection threshold (blue line) was set at mean + 3*mean and a start/end threshold (green line) was set at mean of the amplitude of the channel activity during sleep. The blue and green arrows indicate the start/end and the centre of the detected event, respectively (same in panel A). IV. Only events crossing the detection threshold and whose duration was between 0.5 s and 5 s were considered spindles and further analysed. B) Power spectra was then accounted for every selected event, considering only spindles band frequencies. C) Mean spindles power was then accounted for every channel and Spindles Power Asymmetry (SPA) was then regionally calculated as the ratio between the spindle spectral power of the lesioned and non-lesioned hemisphere.


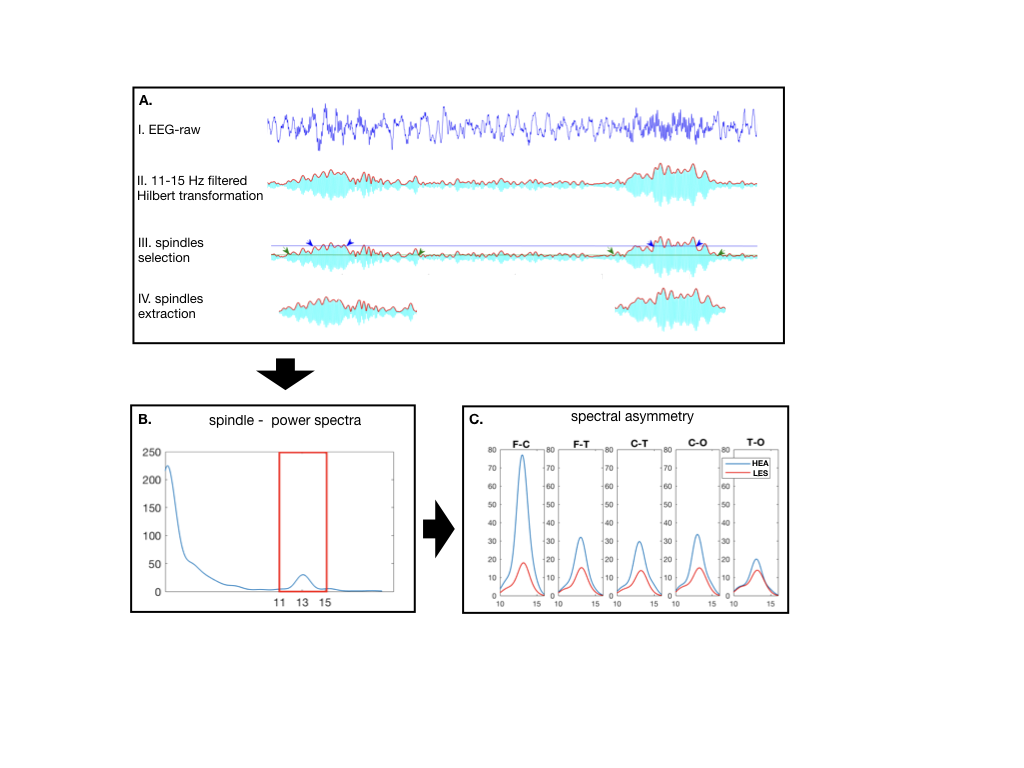

Supplement: Supplementary file 2 — Figure S1: Sleep spindle detection and spindle power asymmetry procedure. [file DMCN-64-1375-s001.docx]
